# The Rattlesnake—Its Poison and Antidote

**Published:** 1874-09

**Authors:** G. M. Rivers

**Affiliations:** South Carolina


VOL. IV.—SEPTEMBER, 1874.—NO. 9.

### Original Communications.

### THE RATTLESNAKE—ITS POISON AND ANTIDOTE.

BY G. M. RIVERS, M.D., OF SOUTH CAROLINA.

The very name produces a thrill of fear, and well it may; for, of all the poisonous serpents of North America, this is undoubtedly the most deadly. And it is very doubtful whether there are any serpents, reptiles or insects whose poison is more quickly and certainly fatal than that of the large rattlesnake of the Carolinas—the Crotalus Horridus; for although there are many species of this serpent, I shall confine myself to this alone. The tribe is peculiar to America, and this species to most parts of North and South America; and in alluding to it as the snake of the Carolinas, I design to write of it only as I know it. In color it is yellow, varying from a bright-yellow to a dark, rusty brown. In fact, so great is the difference of color, that they have been distinguished as highland and lowland. But as the dark and bright are found indifferently in the same localities, the probability is that the difference of color is the difference of sex—the brighter and slimmer snake being the male, the darker and stouter the female. It is yellow, then sometimes very dark, and sometimes bright, with a black stripe or band, the fourth of an inch wide, running spirally from head to tail, and crossing each other in such a way as to form diamonds upon the surface. And so nearly are they the color of the leaves of the forest, where they dwell, that it is difficult to distinguish them unless in motion. They attain a greater size than any of the serpents of the Southern States, nor do I know of any larger in North America—growing sometimes to the length of seven feet and over, and measuring from two to three inches in diameter. By far the largest that has ever come within my personal knowledge, was brought to our village many years ago. This snake was skinned, and the skin filled with sand, which made it somewhat larger than natural. I do not remember the exact measurement, but several gentlemen who saw it at the same time agree with me that it was nine feet long, and over four inches in diameter. One gentleman said "it was as long as a fence-rail, (ten feet), and as large as thát post," pointing to a post five inches in diameter. But these are individual instances of extraordinary growth. The usual growth of a full-grown rattlesnake is from five and a half to six and a half feet in length, and measuring from two to three inches in diameter. This, then, is among the largest of those serpents that take their prey by poisoning. And as they are provided only with poison sufficient in power and quantity to kill quickly those animals upon which they feed, it is natural to suppose that, feeding upon the largest animals, they would require the most deadly poison, and I think experience will hold me out in the opinion. A reptile that can destroy a man in less than an hour is a terrible enemy—and smaller animals in less than a minute; a hen stricken flew twenty yards and was dead—less than a minute, less than a quarter of a minute; and dogs even have been known to die almost instantly.

Dr. Mead, a celebrated English surgeon, who lived during the last century, and devoted much time and study to the poison of insects and reptiles, was of the opinion that the Cobra of India, and the rattlesnake of America, were the most deadly of the serpent tribe. Fortunately for those who live in districts infested with these serpents, they are not aggressive in disposition, and will seldom strike unless provoked or touched. When once aroused, however—and it does not take much to do that—he is the very personification of intense fury; throwing himself instantly in his coil, with eye flashing, tongue vibrating, head flattened and trembling, and rattles ringing incessantly, he is ready for battle, nor can he be intimidated. It is then that a timid man will move daintily about him. But don't think to attack him from behind with safety, for he can throw himself his full length, and somewhat beyond his length, in any direction. In the American Encyclopædia, it is stated "that he cannot, when taken by the hand around the neck, coil himself around the arm, or use any constricting power." This is a great mistake. He cannot only coil himself around the arm, but use power enough completely to paralyze the arm. A negro man taking a large one around the neck about a half mile from home, (which can be easily done with a forked stick), allowed it to coil itself around his arm; finding his arm becoming numbed, he lost his presence of mind, (for he could have uncoiled it with the other hand), and started to run for home, where he arrived barely in time to save himself and get the serpent's head chopped off by a friend. For a while his arm was completely paralyzed. Another mistake from the same source, viz.: that he sounds his rattles some time before striking. This would indeed be a great blessing, but it is not so. Touch him and he strikes instantly, and then sounds his rattles. The fact is, he sounds his rattles when enraged, and as long as he is enraged. These rattles are a unique and singular appendage, formed of hollow, flattened scales of horn, looped on to each other (but not joined together) so that they may be taken apart without breaking. The serpent has one for each year of its life, but in a collection of some fifteen or twenty rattlesnakes, I did not succeed in obtaining one that had over sixteen rattles. I think as the snake grows old, the rattles become brittle and break off, for some very large serpents are killed with very few rattles. These rattles are set in motion by the vibration of the muscles of the tail, and so rapid is it that you cannot perceive the motion. The sound is very much like the shrill notes of the cicada on a hot summer day—not so loud and shrill, and more of a buzzing sound. And the peculiarity of it is that you cannot locate the sound. When a rattlesnake sounds his rattles near you, it appears to be in every direction around you. Great difference of opinion has existed about the use of these rattles, some naturalists supposing that they were intended to warn its enemies—others, to attract its prey; and he no doubt uses them for both purposes. They ring them when enraged or disturbed; they ring them to attract their prey, and perhaps to call their young; for it is very certain that these serpents have the power of attracting to them some, at least, of the animals upon which they feed. They live upon birds, frogs, and other reptiles, which they take generally without the necessity of poisoning; but the principal food are rabbits and squirrels, both of which they attract by the power of fear. When a rattlesnake wishes to take a squirrel which he sees upon a neighboring tree, he goes to the root of the tree and commences to ring his rattles. As soon as the squirrel hears the sound, endowed with instinct that warns him of approaching danger, he becomes intensely excited and alarmed; with bristling hair and chattering incessantly, he continues to run up and down the tree, and even after he sees his enemy, such is the power of fear and the influence of the serpent's eye and appearance, that he continues to advance and recede, coming nearer each time, until he gets in striking distance, when in one instant he is stricken, runs a few yards and drops dead. Some hunters riding along the road saw a squirrel drop suddenly from a tree; they were about to ride up to it to see what was the matter, when one older and more experienced than the rest called out to beware lest a rattlesnake had stricken the squirrel. Upon looking around carefully, they found, as he had said, a large rattlesnake at the foot of the tree, still in his coil.

A laborer, whilst engaged in getting out timber, was attracted by the unusual actions of a large fox squirrel. He was induced to go near, that he might observe it more closely. The squirrel was running up and down the tree, then on the ground a little way, then back again on the tree. It continued this (chattering incessantly, and apparently in great distress) for a long time. Finally, it approached within striking distance of a large rattlesnake, when in one instant the snake had stricken it, and taking it back of the neck, wound itself quickly around it, and commenced the slow and tedious operation of swallowing it. The laborer, induced by curiosity, waited to see the process, and so admirably adapted is the head and neck of this serpent for the purpose, that it can swallow an animal much larger than itself. The symphysis of the lower jaw is not united by bone, but by a ligament capable of great distention. It has a row of small, sharp teeth on each side of the jaws, which are very wide apart at the base, and without bony union. This arrangement enables it to hold on with one side of the lower jaw, whilst the other side is advanced forward to take a fresh hold. The throat is capable of almost indefinite distention, and whilst engaged in this operation a thick, lubricating saliva pours out abundantly. This, then, with the power of suction, enables it to perform such astonishing feats of deglutition.

One word with regard to the trail of this serpent. It is straight, whilst that of other serpents is zigzag. He moves with a perpendicular motion; other serpents with a horizontal; and so his trail may be distinguished from other serpents, when observed in crossing a road.

The deadly fangs are situated in the upper jaw, one on each side, and when not in use, lay flat against the roof of the mouth, covered by a thin curtain-like membrane. These fangs are not fixed in bony sockets, but moved by strong muscles, and cannot go beyond the perpendicular; as they are erected, the base presses upon the sack of poison, which is injected through a hollow extending from the base to near the point, which is exceeding sharp. These fangs are curved, and are much the form and shape of the talons of a bird of prey. This poison is injected through the fangs with considerable force, so that when examining the head of a snake, care should be taken lest in pressing the fangs down upon the sack of poison, the poison might be injected upon the hands at the distance of a couple of feet or more. A young man of my acquaintance, whilst examining the head of a snake, had the poison squirted upon his hands, which, having sores upon them, gave him much uneasiness. But he sucked off the poison immediately, and escaped without any unpleasant effects. His tongue and teeth were stained black by it. At the root of the large fangs, and loosely attached to the gums, are found several rudimentary fangs, ready to take the place of the old ones and commence to grow, should these latter by any accident become broken off or drawn out, which is easily done when the animal strikes a hard substance. When made to strike a soft piece of wood, he is apt to leave one or both fangs in it. This serpent is viviparous, the young being born alive; and what is most singular, when very young, they are carried in the stomach of the mother, and perhaps nourished by the half-digested food found there. Many well-authenticated instances where the young have been seen to go down the mother's throat, have come to my knowledge; but one or two will suffice. A gentleman saw a large rattlesnake sunning itself upon a log, and near it a number of young ones about three inches long. (They produce from eighteen to twenty at a birth.) He flung at it for the purpose of alarming it, when immediately she rung her rattles, and approaching her young with her mouth wide open, they went home with a rush. Some negro laborers, whilst repairing an old dam, discovered a very large rattlesnake, and near her, in the sun, her young ones. As soon as she was disturbed, she approached her young with her mouth wide open, when they ran quickly down her throat.

I remarked before that the poison was injected with considerable force, and it is probable that as much as ten drops or more, depending upon the size of the snake—for the larger the snake, the larger the sack of poison, and the greater the danger from the bite—(a snake a year old having a rattle and a button)—produces but slight effect. Perhaps, also, as the snake gets older and larger, the poison becomes more concentrated, and is therefore not only greater in quantity, but more deadly in effect. I said about ten drops was injected through each tooth by a full-grown serpent. These fangs being curved, when the animal strikes, they are inserted between the skin and flesh, in the cullular tissue, where it can escape freely in all directions. Were they straight, they would be driven into the muscles, the contraction of which would prevent the flow of the poison. The poison is soon exhausted and after the animal has stricken several objects in succession, it ceases to produce death. Three half-grown hogs stricken in succession by the same serpent, the first died instantly, the second in a short time, and the third recovered. This poison is a greenish viscid substance, and is supposed not to be poisonous when taken by the mouth. I think that has not been tested. Dr. Mead, the English surgeon alluded to, tried half a drop, and another physician a whole drop. Both escaped without any ill effect whatever. But this was no test, for this substance is a poison and not a virus, and requires a certain quantity to kill. If my opinion is correct that twenty drops are injected at once, then one drop would be no test. The fact that the animal takes into its stomach the food it had poisoned, might be proof, because when made to strike itself it instantly destroys its own life. The effect of the poison when a human being is stricken is an intense burning in the wound, extending over the body, even to the top of the head; vertigo, nausea and vomiting, dimness of vision, irregular spasmodic action of the muscles; then delirium, relaxation, depression of the vital powers, and death by syncope. It is, then, a narcotic sedative, and the remedy must be stimulants. In proposing this treatment, I do not pretend to have discovered anything new, but only to urge upon the public the only reliable remedy, viz.: alcoholic stimulants.

As soon, then, as a person is bitten, let a ligature, a hard, strong string, be tied six inches above the wound; this should be done immediately. Then, with a sharp knife cut entirely through the skin over and through the wound where each tooth enters. Then suck out the poison, extract it with cups, or apply a chicken recently killed and split in half, the flesh side to the wound. As soon as this is done, move the string a little higher, using a softer string drawn not quite so tightly. The object is to retard the absorption of the poison remaining in the system, and allow it to be as slowly and gradually impressed upon the nervous centre as possible. As soon as it possibly can be obtained, (for you have only a few moments to act, and whatever is to be done must be done quickly), give the whisky and give it freely—a gill every twenty minutes until the patient is thoroughly intoxicated, and keep him so for twenty-four hours. If this plan is pursued with promptness and energy, few patients will die. And now yearly some life is lost in those localities where this reptile abounds. But, after all we may do, when an old and large rattlesnake strikes a person, a child particularly, in a part favorable for rapid absorption, recovery is doubtful.

A few cases illustrative of the above will suffice. A negro child seven years old, whilst sitting upon the rude steps to a temporary summer residence and eating his breakfast, was attracted by a bright object under him, and struck at it with his spoon; his mistress sitting near by and seeing him make a wry face, said to him, "What is the matter now?—come here." He answered, "Scorpion bite me," and got up to go to her. Before reaching her, he vomited; his eyes were glazed and drawn back with a violent spasm; would have fallen, had he not been supported by a servant standing near; even then, his teeth were so firmly clenched that a spoon could not be introduced between them. Squatting now upon his hands, he hopped about the room in a singular manner, with his eyes closed, apparently perfectly unconscious of all around him, noticing nothing and saying nothing, but answering faintly when called. He continued this some time, then became quite exhausted; his lower jaw was relaxed and hanging down; his skin looked as if it had been oiled. He lay perfectly quiet, complaining of no pain, and died very easily three hours after he was stricken. As soon as the accident occurred, a servant was ordered to go for the Doctor. Whilst saddling the horse near the door, he saw a large rattlesnake, with eleven rattles, crawling from under the house, the author of the terrible tragedy. Such was the confusion and excitement attending this accident, that although much was done for the child, a little pet, yet no efficient remedy was used. But such, I think, was the rapid diffusion of the poison, that nothing could have saved his life. In less than three minutes from the occurrence of the accident, he was stricken with death.

Another case was that of a country lad who, whilst plowing in a field, was attracted by the cry of dogs in pursuit of a cat. Having his gun with him, he ran to the edge of the wood, and was in the act of crossing a low worm fence, when he was stricken upon the heel (he was barefoot.) Instantly, as if by inspiration, he drew a buckskin string from his pocket and tied it tightly around his leg. Soon after, the hunters coming up, gave him what whisky they had with them—about a pint. By this time he was much affected by the poison—suffering with vertigo, nausea and vomiting, dimness of vision, and prostration of strength. They took him up and carried him to a vacant house near by, and sent for his brother, who, being a man of practical good sense, plied him well with whisky. He was totally unused to it, but he took in the course of the night about three pints. Next morning I was sent for to visit him, and found him entirely relieved from the effect of the snake-bite, but suffering somewhat with "big-head," caused by the lively time he had had with the whisky. This was a large serpent, measuring five and a half feet, and having nine rattles.

A son of Mr. W., whilst at a log-rolling, was stricken by a large rattlesnake—I could not ascertain the number of rattles. The boy was nine years old. In less than twenty minutes, he was made to swallow one pint of whisky (enough to have killed him, had it not been antidoted by the poison.) He became quite easy, slept soundly, and next day was well.

Young B., fourteen years old, was stricken by one of the largest, he said, he ever saw, whilst in company with his brothers getting lightwood. They immediately tied a ligature very tightly above the wound, put him in the wagon and carried him home. They got him drunk as soon as possible with large doses of whisky, and the next day he was well enough to go in search of the snake—which, strange to say, he did not find.

